# Supplementary material for: Spatiotemporal pattern of land use and land cover changes of upper Sangu-Matamuhuri Watershed in the South-Eastern Bangladesh
Source: PLoS One. 2025 Jul 2;20(7):e0327284. doi: 10.1371/journal.pone.0327284 (PMC12221057; doi:10.1371/journal.pone.0327284)
Supplement: Attachment — Submitted filename: Reviewer Report.docx [file pone.0327284.s007.docx]

**Reviewer Report: Abstract**

**Manuscript Title**:
*Spatiotemporal changing pattern of land use and land cover of Sangu-Matamuhuri watershed in the South-Eastern part of Bangladesh*

**Major Comments:**

1. **Clarity and Focus**:
   - **Comment**: The abstract provides a good overview of the methods used and the results obtained. However, the main research question or objective is somewhat hidden. While it is implied that the objective is to assess land use and land cover (LULC) changes, it would benefit from a clearer and more explicit statement of the research aim at the beginning of the abstract.
   - **Recommendation**: Consider restructuring the opening sentence to explicitly state the purpose of the study, for example: *"This study aims to analyze the spatiotemporal patterns of land use and land cover (LULC) changes in the upper part of the Sangu-Matamuhuri Watershed (SMW) from 1988 to 2019 using integrated remote sensing (RS) and geographic information system (GIS) approaches."*
2. **Overload of Technical Details**:
   - **Comment**: While technical terms like "Geo-spatial object-based supervised classification" and accuracy statistics (e.g., Kappa coefficient, classification accuracy) are important, their inclusion in the abstract seems to clutter the flow and may overwhelm readers unfamiliar with such details. Abstracts should provide an overview, not the full technical breakdown.
   - **Recommendation**: Consider simplifying or omitting some of the more technical terms (such as "object-based supervised classification") and instead focus on the broader methodological approach (i.e., remote sensing and GIS-based analysis). You could move the accuracy metrics to the results section of the main manuscript and focus more on the implications of the findings in the abstract.
3. **Impact and Context**:
   - **Comment**: The findings related to forest loss, agricultural expansion, and water body reduction are well-presented, but the abstract could benefit from a stronger statement about the broader ecological and social impact of these changes. The mention of "serious threat to watershed resources and ecosystem services" is important but could be further emphasized.
   - **Recommendation**: Strengthen the closing statement by clearly linking the LULC changes to specific environmental and social consequences, such as biodiversity loss, water scarcity, and implications for local communities. For example: *"These LULC changes not only threaten ecosystem services but also pose significant risks to local communities dependent on these resources for livelihood and water supply."*

**Minor Comments:**

1. **Typographical and Grammatical Issues**:
   - **Comment**: There are some minor grammatical issues. For example, in the sentence "So. the findings of this study indicate important policy implications," the period after "So" should be a comma, and the word "So" may not be the best way to start the sentence in a formal abstract.
   - **Recommendation**: Replace "So" with "Therefore," or rephrase the sentence: *"The findings of this study have important policy implications for sustainable watershed management..."*
2. **Inconsistent Use of Units**:
   - **Comment**: In some places, the units of measurement (e.g., hectares) are abbreviated (ha), while in others, "hectares" is spelled out (e.g., "234634 ha" vs. "21787 hectares"). Consistency is important in maintaining the professional tone of the manuscript.
   - **Recommendation**: Ensure consistent use of units throughout the manuscript, including the abbreviation of "hectares" as "ha" for uniformity.
3. **Sentence Length and Readability**:
   - **Comment**: Some sentences are quite long and could be broken into shorter, more digestible segments. For example, the sentence starting with "Conversion results obtained from temporal overlay operation..." is quite dense.
   - **Recommendation**: Break long sentences into shorter parts for better readability. For instance, you could split the sentence about "conversion results" into two: *"The temporal overlay operation revealed that forest patches were converted to shrubland due to deforestation and forest burning. This shrubland was then cleared or re-burned for shifting cultivation, significantly increasing agricultural land."*

**Summary Recommendation:**

- **Overall Recommendation for Abstract**:
  The abstract provides a solid foundation by summarizing the methods and key findings. However, improvements in clarity, readability, and impact are necessary to make it more engaging and accessible to a broader audience. Specifically, the research objective should be more clearly stated at the beginning, and technical details should be simplified. The implications of the study should be more forcefully conveyed to highlight the importance of the findings.

**Reviewer Report: Introduction**

**Major Comments:**

1. **Clarity and Focus**:
   - **Comment**: The introduction effectively sets the stage for the study by discussing the importance of forests, the consequences of land use and land cover (LULC) changes, and the impact on watershed areas. However, the flow of ideas could be more structured. The section starts broadly by discussing forests and LULC, but jumps to specific issues in Bangladesh (e.g., water scarcity in the Sangu-Matamuhuri Watershed) and then back to global LULC research. This could confuse readers about the specific focus of the paper.
   - **Recommendation**: Consider organizing the introduction more clearly by separating the global context of LULC changes, the case of Bangladesh and the Sangu-Matamuhuri Watershed, and the significance of the research. For example:
     1. General importance of forests and LULC changes globally.
     2. Specific issues in Bangladesh, especially the Sangu-Matamuhuri Watershed.
     3. Previous studies and gaps in knowledge.
     4. The objectives of the current study.
2. **Relevance of Literature Review**:
   - **Comment**: While the introduction reviews relevant literature on LULC changes and watershed management, some cited studies seem tangential or not directly related to the research focus (e.g., studies on socio-economic determinants affecting landscape restoration). Additionally, certain key studies (e.g., those directly related to LULC changes in Bangladesh) should be highlighted more prominently.
   - **Recommendation**: Streamline the literature review to focus more on studies that directly relate to LULC changes and watershed management, especially in Bangladesh. Ensure that the reviewed literature builds a clear rationale for why this study is needed and how it fills a gap in existing knowledge.
3. **Research Gap and Justification**:
   - **Comment**: The introduction mentions that "little is known about LULC change dynamics of Sangu-Matamuhuri watershed." While this is an important statement, it should be expanded to clearly articulate why this gap is significant and how the current study will address it. For example, how does filling this gap contribute to broader environmental or policy goals in the region?
   - **Recommendation**: Strengthen the justification for the study by clearly stating the societal, environmental, and policy implications of better understanding LULC changes in this watershed. A stronger argument for the importance of this research will engage readers more effectively.
4. **Study Objectives**:
   - **Comment**: The study’s objectives are clearly stated at the end of the introduction, but they could be more specific. For instance, it is not clear what "nature, extent, and pattern" of LULC changes will be analyzed or how these findings will be used to inform watershed management.
   - **Recommendation**: Clarify the objectives by specifying the types of LULC changes that will be studied (e.g., forest to agriculture, settlement growth) and how these changes will be quantified or analyzed. Additionally, briefly mention how the findings could contribute to practical conservation or management efforts.

**Minor Comments:**

1. **Typographical and Grammatical Issues**:
   - **Comment**: There are a few minor grammatical issues throughout the text. For example, in the sentence "Forests are the natural heart of an environment that plays a vital role in not only reserving wild animals, and wood-timber generation purposes but also balancing micro-climate, local economy-ecology, and sub-surface biota," the phrase "wood-timber generation purposes" is awkward, and "not only... but also" is not properly parallel.
   - **Recommendation**: Rephrase to improve clarity and grammar. For example: *"Forests play a vital role in preserving wildlife, generating wood and timber, and balancing the micro-climate, local economy, and subsurface biota."* A thorough proofreading is recommended to catch similar issues.
2. **Reference Formatting**:
   - **Comment**: The references are inconsistently formatted. Some citations use numbers (e.g., "[1]"), while others use author names and years (e.g., "Barua and Haque 2013"). This inconsistency can be confusing to the reader and does not adhere to standard academic citation formats.
   - **Recommendation**: Ensure all references are formatted consistently according to the journal’s guidelines (either numbered or author-date format). If using numbered citations, remove author names and years from in-text citations.
3. **Sentence Length and Readability**:
   - **Comment**: Several sentences are quite long and complex, making them difficult to follow. For example, the sentence starting with "Silt accumulation hampered navigation and led to the extinction of many fish species..." could be more concise.
   - **Recommendation**: Break longer sentences into shorter, more manageable ones. For example: *"Silt accumulation has hampered navigation and led to the extinction of many fish species. More than 80% of the Bandarban population, who depend on the rivers for water, now face acute water shortages due to the drying of the rivers."*
4. **Use of Terms**:
   - **Comment**: The term "selfishness" in the phrase "selfishness in terms of taking advantage of natural resources" seems subjective and unscientific.
   - **Recommendation**: Consider rephrasing to use more neutral and objective language. For example: *"The unsustainable exploitation of natural resources..."*
5. **Transition to Objectives**:
   - **Comment**: The transition from the literature review to the study objectives is somewhat abrupt. After discussing general LULC research, the study objectives are stated without a clear transition.
   - **Recommendation**: Add a short transition sentence before the objectives, summarizing the research gap and leading into the study’s aims. For example: *"Despite these advancements in LULC research, there remains a lack of comprehensive spatiotemporal analysis of LULC changes in the Sangu-Matamuhuri Watershed. Therefore, this study aims to..."*

**Summary Recommendation:**

- **Overall Recommendation for Introduction**:
  The introduction provides a strong foundation by discussing the importance of forests, LULC changes, and the specific challenges faced by the Sangu-Matamuhuri Watershed. However, the flow of ideas could be improved for better readability and coherence. The literature review should be more focused on directly relevant studies, and the research gap and objectives need to be more clearly articulated. Additionally, proofreading for minor grammatical and stylistic issues is recommended.

**Reviewer Report: Materials and Methods**

**Major Comments:**

1. **Study Area Description**:
   - **Comment**: The description of the study area is informative but could benefit from further contextualization. While geographic coordinates, elevation, and population data are provided, there is limited discussion about why this region is ecologically or hydrologically significant, especially in relation to land use and land cover (LULC) changes.
   - **Recommendation**: Expand the description of the study area to include more information on the ecological importance of the upper part of the Sangu-Matamuhuri Watershed (SMW). For example, why is this area prone to deforestation? What are the main environmental challenges? You also mention tourism, but it would be useful to elaborate on how this economic activity interacts with land use practices.
2. **Data Acquisition and Image Preprocessing**:
   - **Comment**: The section on data acquisition and image preprocessing provides good detail about how the satellite images were selected and processed. However, the section could be clearer regarding the reasoning behind some decisions, such as the selection of specific Landsat images and the choice of analysis periods (1988, 1999, 2009, and 2019).
   - **Recommendation**: Add a brief explanation of why these specific years were chosen for analysis. Was it due to data availability, or do these years represent significant periods of environmental change in the region? Additionally, clarify why some images were excluded, such as the image from 1999 that was replaced with one from February 2000.
3. **Field Sampling and Classification Algorithm**:
   - **Comment**: The section on the classification algorithm is comprehensive but somewhat dense. While the object-based image classification approach is clearly explained, the rationale for choosing this method over others (e.g., pixel-based classification) could be more explicitly stated.
   - **Recommendation**: Provide a brief justification for the use of object-based classification over pixel-based classification, perhaps with reference to the specific characteristics of the study area (e.g., heterogeneous land cover, complex terrain). Additionally, clarify the significance of the selected scale parameters (e.g., scale, shape, compactness), as this will help readers unfamiliar with the technical details understand why these values were chosen.
4. **Accuracy Assessment**:
   - **Comment**: The accuracy assessment section is well-detailed, but the explanation of the error matrix and accuracy metrics could be simplified for clarity. Additionally, the reasoning behind the selection of 30 GPS points for accuracy assessment is not clear.
   - **Recommendation**: Simplify the explanation of the error matrix and the related accuracy metrics. For example, you could briefly explain the purpose of each metric (producer's accuracy, user's accuracy, overall accuracy, and Kappa coefficient) in non-technical terms before presenting the equations. Also, explain why 30 GPS points were used for accuracy assessment—was this based on field conditions, image coverage, or other factors?
5. **LULC Change Analysis**:
   - **Comment**: The methodology for LULC change analysis is well-explained but could benefit from a more structured presentation. For instance, the steps for calculating magnitude change, percentage change, and annual rate of change are useful, but the equations could be more clearly separated from the text for better readability.
   - **Recommendation**: Consider formatting the equations in a more distinct and structured way (e.g., using bullet points or separate lines) to improve readability. Additionally, provide more detail about how the change matrix was used to derive insights into land use transitions (e.g., forest to agriculture, shrubland to settlement). A brief explanation of how the change matrix informs land management decisions would also add value.

**Minor Comments:**

1. **Typographical and Grammatical Issues**:
   - **Comment**: There are a few minor grammatical issues and awkward phrasings in the text (e.g., "The study area was acquired using satellite imagery..."). In some places, the text could be more concise and clearer.
   - **Recommendation**: Proofread the entire section for minor grammatical errors and awkward phrasing. For example, the phrase "The study area was acquired using satellite imagery" could be reworded to: *"Satellite imagery was used to delineate the study area."* Similar improvements could be made throughout.
2. **Reference Formatting**:
   - **Comment**: There are inconsistencies in the citation format. For example, some references use numbers (e.g., [41]), while others use author names (e.g., "Robertson and King, 2011").
   - **Recommendation**: Ensure that all references are formatted consistently according to the journal’s specific style guide. If using numbered citations, avoid including author names in the text.
3. **Table and Figure References**:
   - **Comment**: You mention several tables and figures (e.g., Fig 1, Fig 2, Table 1, Table 2), but the text could benefit from more direct and descriptive references to these figures and tables. For example, instead of just saying "as shown in Fig 1," you could briefly describe the content of the figure.
   - **Recommendation**: Provide more descriptive references to figures and tables. For example: *"The study area, as shown in Fig 1, includes the upper part of the Sangu-Matamuhuri Watershed in the southeastern region of Bangladesh."* This helps guide the reader more effectively.
4. **Methodological Workflow**:
   - **Comment**: The methodological workflow is summarized in Fig 3, but this figure is not discussed in detail in the text.
   - **Recommendation**: Include a brief description of the methodological workflow in the text to give readers an overview of the steps involved in the analysis. For example: *"The methodological workflow (Fig 3) outlines the steps from data acquisition and preprocessing to classification and accuracy assessment."*

**Summary Recommendation:**

- **Overall Recommendation for Materials and Methods**:
  This section provides a thorough and detailed explanation of the methods used for LULC analysis in the Sangu-Matamuhuri Watershed. However, some areas require clarification and improvement to enhance readability and flow. The study area description could be expanded to provide more context about the ecological importance of the region. Additionally, the rationale for certain methodological choices (e.g., the use of object-based classification, the selection of specific years, and the number of GPS points) should be more clearly explained. Minor grammatical issues and reference formatting inconsistencies should also be addressed.

**Reviewer Report: Results**

**Manuscript Title**:
*Spatiotemporal Changing Pattern of Land Use and Land Cover of Sangu-Matamuhuri Watershed in the Southeastern Part of Bangladesh*

**Major Comments:**

1. **Mapping Accuracy and LULC Change Status:**
   - **Comment**: The accuracy assessment for LULC classification is comprehensive, providing essential metrics such as overall accuracy, kappa statistics, producer's accuracy, and user’s accuracy for each class. These results are crucial for validating the reliability of the classification maps.
   - **Recommendation**: The accuracy discussion could be expanded to include a brief explanation of why certain classes, such as *shrub land*, had lower user accuracy in 1988 (70%) compared to other years. Moreover, it would be beneficial to discuss whether any specific challenges or confusion between classes (e.g., shrub and forest) could explain variability in accuracy across different periods.

Additionally, the text would benefit from introducing the importance of the Kappa statistic before presenting the results. A brief explanation of what constitutes a "good" or "high" kappa value (e.g., above 0.80 is generally considered very good) would help less familiar readers contextualize the significance of these values.

2. **LULC Change Analysis:**
   - **Comment**: The results clearly outline the spatial-temporal changes in LULC classes, with forest land showing a continuous decrease and agricultural land showing a significant increase. While the results are well-presented, the explanation could benefit from more in-depth analysis regarding the drivers behind these changes.
   - **Recommendation**: Provide a more detailed analysis of the potential drivers behind the significant LULC changes observed in the study area. For example, the conversion of forest land to agriculture and settlements is attributed to population growth and upland cultivation, but it would be useful to expand on other contributing factors, such as government policies, economic incentives, or infrastructural development in the region. Additionally, consider discussing the environmental implications of such trends, especially the impact of deforestation and agricultural expansion on biodiversity and water resources.
3. **Table 4 (Areal Distribution of LULC Classes):**
   - **Comment**: Table 4 outlines the areal distribution of LULC classes across the study periods, which is essential for understanding the extent of land cover change. While the numbers are informative, the table could be further enhanced with more insight into the observed trends.
   - **Recommendation**: In the text accompanying Table 4, provide a concise summary of the key trends, such as the overall percentage decrease in forest cover from 1988 to 2019 (from 76% to 54%) and the corresponding increase in agricultural land (from 7% to 25%). Additionally, consider discussing whether these changes align with regional or national trends in land use changes, drawing comparisons with other studies if available.
4. **Change Matrices and Visual Representation (Figs. 7–10):**
   - **Comment**: The change matrices presented in Figures 7, 8, 9, and 10 offer valuable insights into the transitions between different LULC classes over time. However, the interpretation of these matrices could be elaborated upon to better explain the significance of the conversions.
   - **Recommendation**: Expand the discussion of the change matrices to explain the ecological and socio-economic significance of the observed conversions. For instance, the substantial conversion of forest land to agriculture should be discussed in relation to its long-term sustainability and potential consequences for soil degradation, water resources, and local communities. Similarly, the conversion of shrubland into other LULC classes could be contextualized with respect to the shifting land management practices in the region.

Additionally, consider highlighting the most significant transitions in a summary table or diagram, which would make it easier for readers to grasp the key findings from the change matrix.

5. **Overall Gain and Loss (Table 5):**
   - **Comment**: Table 5 effectively summarizes the gain and loss percentages of LULC classes over the study periods. However, the discussion around these results could be enriched by drawing connections to broader environmental and policy implications.
   - **Recommendation**: In the section discussing Table 5, consider elaborating on the broader implications of these gain and loss patterns. For example, the loss of 31.21% of forest cover between 1988 and 2019 could be linked to discussions on climate change, carbon sequestration, and the role of forests in regulating water cycles. Similarly, the significant gain in agricultural land (22.77%) could be discussed in the context of food security, land tenure issues, or agricultural policy in Bangladesh.

Furthermore, consider including a brief discussion on the potential long-term sustainability of these trends. Is the continuous expansion of agriculture at the expense of forests and shrublands sustainable, given the environmental consequences?

**Minor Comments:**

1. **Typographical and Grammatical Issues**:
   - **Comment**: There are minor grammatical errors and awkward phrasings throughout the text, such as "The area of water body was found gradually decreased...".
   - **Recommendation**: Proofread the text to correct minor grammatical issues. For example, rephrase sentences like "The area of water body was found gradually decreased..." to "The area of water bodies gradually decreased...".
2. **Figure Citations**:
   - **Comment**: There are several instances where figures are referenced, such as Fig 4, Fig 5, and Fig 6, but the references could be more descriptive.
   - **Recommendation**: Instead of simply stating "Fig 4 shows the multi-temporal LULC maps," provide more context such as: *"Fig 4 displays the multi-temporal LULC maps, illustrating the spatial distribution of forest, agriculture, shrubland, settlements, and water bodies across the years 1988, 1999, 2009, and 2019."* This helps the reader understand the content of the figure before viewing it.
3. **Numerical Consistency and Formatting:**
   - **Comment**: In some areas, numbers are not formatted consistently, particularly with the use of commas (e.g., "310317" vs. "310,317").
   - **Recommendation**: Ensure consistent formatting of numbers throughout the manuscript. Use commas for large numbers greater than 1,000 to improve readability (e.g., "310,317" instead of "310317").
4. **Equation References:**
   - **Comment**: The equations used in the LULC change analysis (e.g., Eq. vi, vii, viii) are not referenced in this section, but are crucial for understanding how changes were calculated.
   - **Recommendation**: Consider briefly referring back to the equations introduced in the methodology section when discussing the results of the change analysis. For example: *"The magnitude and percentage changes were calculated using equations (Eq. vi and vii) as described in the methods section."*
5. **Discussion of Water Body Changes:**
   - **Comment**: The decrease in water bodies is mentioned, but the potential causes are only briefly touched upon (e.g., withdrawal of water for cultivation).
   - **Recommendation**: Expand on the possible reasons for the reduction in water bodies, and discuss the implications for water availability, hydrology, and local ecosystems. Consider linking this to broader discussions of water management challenges in the region, particularly in the context of climate change and agricultural expansion.

**Summary Recommendation:**

- **Overall Recommendation for Results**:
  The results section is thorough and presents key findings on the temporal changes in LULC within the Sangu-Matamuhuri watershed. However, several areas could be improved to enhance the clarity and depth of the analysis. The accuracy assessment is strong, but the rationale behind certain accuracy variations could be better explained. The LULC change analysis would benefit from a more detailed discussion of the underlying drivers and environmental consequences of the observed trends. Additionally, the change matrices are informative, but the interpretation could be expanded to provide deeper insights into the significance of land use transitions. Minor grammatical issues and formatting inconsistencies should also be addressed.

**Reviewer Report: Conclusion**

**Major Comments:**

1. **Summary of Key Findings**:
   - **Comment**: The conclusion provides a good summary of the major LULC changes observed in the study area, particularly the decrease in forest land and the corresponding increase in agriculture, shrubland, and settlement areas. However, the discussion could be more explicitly linked to the broader implications of these changes, both environmentally and socio-economically.
   - **Recommendation**: Consider expanding the conclusion to emphasize the broader implications of the findings. For instance, the reduction in forest cover and increase in agriculture could be discussed in the context of regional deforestation trends, climate change, and biodiversity loss. Similarly, the drying of water bodies could be connected to water scarcity issues, particularly in light of changing rainfall patterns due to climate change. Highlighting these broader concerns would help emphasize the urgency of the situation and justify the need for reforestation and sustainable land management practices.
2. **Linking Results to Ecosystem Services**:
   - **Comment**: The conclusion mentions that LULC changes pose a threat to the ecosystem services of the Sangu-Matamuhuri Watershed (SMW), but this point could be elaborated further. Specifically, the conclusion could more clearly articulate how the observed changes are affecting the provision of key ecosystem services such as water regulation, soil conservation, and carbon sequestration.
   - **Recommendation**: Expand on the discussion of ecosystem services by specifying which services are most at risk due to the LULC changes. For example, the reduction in forest cover likely compromises the watershed's ability to regulate water flow, prevent soil erosion, and store carbon. Similarly, the drying of water bodies affects both water availability and the local biodiversity that depends on these aquatic habitats. By making these connections explicit, the conclusion would present a more comprehensive understanding of the ecological consequences of the observed LULC changes.
3. **Recommendations for Future Actions**:
   - **Comment**: The conclusion appropriately suggests reforestation and stopping forest conversion as necessary actions to mitigate the negative effects of LULC changes. However, the recommendations could be more specific, particularly regarding how these measures could be implemented.
   - **Recommendation**: Strengthen the recommendations by providing more concrete suggestions on how reforestation and sustainable land management practices can be achieved. For instance, mention specific reforestation techniques (e.g., agroforestry, replanting of native species) and discuss potential sources of funding or governance models that could support these efforts. Additionally, the suggestion to develop alternative income sources for forest-dependent communities is important but could be expanded to include examples of such alternatives (e.g., eco-tourism, sustainable agriculture, or non-timber forest products). This would make the recommendations more actionable and grounded in practical solutions.
4. **Water Resources and Community Impacts**:
   - **Comment**: The conclusion highlights the reduction in water bodies and its impact on indigenous communities and irrigation. This is an important point, but it could be expanded to include more details on the socio-economic consequences of water scarcity for local populations.
   - **Recommendation**: Expand on the discussion of water scarcity and its potential impacts on local communities. For example, how might the drying of water bodies affect agricultural productivity or the availability of drinking water for indigenous peoples? Consider discussing the long-term socio-economic impacts, including the potential for increased migration or conflicts over water resources. Additionally, mention potential strategies for water conservation or the development of alternative water sources (e.g., rainwater harvesting, improved irrigation techniques).
5. **Urgency and Long-Term Outlook**:
   - **Comment**: The conclusion touches on the long-term consequences of LULC changes, such as water shortages and increased flooding. However, the sense of urgency and the potential long-term outlook could be more strongly emphasized.
   - **Recommendation**: Strengthen the conclusion by emphasizing the urgency of addressing LULC changes in the SMW. For example, mention the potential for irreversible ecological damage if current trends continue and the importance of immediate intervention to prevent further degradation. Discuss the long-term risks associated with climate change, deforestation, and land degradation, and the need for comprehensive land-use planning that balances environmental sustainability with economic development.

**Minor Comments:**

1. **Clarity and Flow**:
   - **Comment**: The flow of the conclusion could be improved, particularly in terms of transitioning between different points. For example, the transition from discussing forest loss to discussing water bodies feels somewhat abrupt.
   - **Recommendation**: Improve the flow of the conclusion by using clearer transitions between sections. For instance, after discussing forest loss, you could introduce the topic of water bodies by saying, *"In addition to the loss of forest cover, the study also revealed a concerning trend of water body reduction, which poses a critical challenge for water availability in the region."* This would help create a more cohesive narrative.
2. **Consistency in Data Presentation**:
   - **Comment**: The conclusion repeats some of the numerical findings (e.g., forest land decreased from 234,634 ha to 168,476 ha), but these numbers have already been presented in the results section. While it is important to summarize key findings, the repetition could be reduced.
   - **Recommendation**: Consider summarizing the LULC changes more concisely, rather than repeating the exact figures from the results section. For example, you could say, *"The study revealed a significant decrease in forest cover, with a substantial portion being converted into agricultural land."* This would avoid redundancy while still conveying the key trends.
3. **Use of Technical Terminology**:
   - **Comment**: The term "LU" (land use) is used in the conclusion without being fully explained. While this may be clear to some readers, it could be confusing for those unfamiliar with the abbreviation.
   - **Recommendation**: Avoid using abbreviations like "LU" without explanation. Instead, use the full term "land use" to maintain clarity. This will ensure that the conclusion is accessible to a broader audience.
4. **Typographical and Grammatical Issues**:
   - **Comment**: There are some minor grammatical issues in the conclusion, such as the sentence: *"A vast area of forest land was transformed into agriculture in the form of shifting cultivation, mixed orchard, cash crop cultivation, etc. caused soil erosion, and quick runoff during rains resulting in flash floods and landslides."*
   - **Recommendation**: Revise sentences for clarity and grammar. For example, the sentence above could be rephrased as: *"A vast area of forest land was transformed into agriculture, including shifting cultivation, mixed orchards, and cash crop cultivation. This land-use change has caused soil erosion and rapid runoff during rains, leading to flash floods and landslides."* Similarly, proofread the entire conclusion for other minor grammatical issues.

**Summary Recommendation:**

- **Overall Recommendation for Conclusion**:

The conclusion provides a solid summary of the key findings from the study, particularly the significant changes in forest cover and agricultural expansion in the Sangu-Matamuhuri Watershed. However, the conclusion could be strengthened by:

- Expanding the discussion of the environmental and socio-economic implications of LULC changes.
- Providing more concrete and actionable recommendations for reforestation and sustainable land management.
- Emphasizing the urgency of addressing these changes to prevent long-term ecological damage.

Additionally, the overall flow of the conclusion could be improved, and minor grammatical and typographical issues should be addressed. With these revisions, the conclusion will be more impactful and provide a clearer call to action for the preservation of the SMW’s natural resources.

**The Summary of the Manuscript based on your criteria stated :**

**1. Reading the Manuscript Thoroughly**

- **ResearchQuestion**:
  The manuscript aims to assess and monitor the changes in Land Use and Land Cover (LULC) in the Sangu-Matamuhuri Watershed (SMW) between 1988 and 2019, highlighting the spatial-temporal patterns of LULC classes such as forest, agriculture, shrubland, settlement, and water bodies. The research question is clear and addresses the critical issue of land-use changes over time, which has significant environmental and socio-economic implications.
- **Methodology**:
  The methods used (satellite imagery classification, accuracy assessments, and change matrices) are appropriate for LULC change detection. The manuscript adequately explains the process of image classification, accuracy assessment, and the use of change matrices to evaluate the transitions between LULC classes over the study period.
- **Results**:
  The results are comprehensive, with detailed analysis of the changes in LULC classes over time. The manuscript provides a clear breakdown of the changes in forest cover, agricultural land expansion, and other LULC categories. The use of tables, figures, and change matrices adds significant value to the presentation of the results.
- **Conclusions**:
  The conclusions summarize the key findings effectively but could be expanded to discuss broader environmental and socio-economic implications of these changes. Recommendations for reforestation and stopping forest conversion are relevant but could be further detailed.

**2. Assessing the Overall Quality**

- **Originality**:
  The research is **moderately novel**, as it addresses LULC changes in a specific region (Sangu-Matamuhuri Watershed) that may not have been widely studied before. However, LULC change detection is a well-established field, so the novelty lies in the specific geographic focus and the temporal scope (1988-2019). The study contributes valuable data on forest degradation, agricultural expansion, and water body reduction in this region, which could inform future land management and conservation efforts.
- **Relevance**:
  The work is **highly relevant** to the field of environmental studies, geography, and land-use management. It fits well within the scope of journals that focus on land cover changes, environmental degradation, and watershed management. The findings have practical implications for land management, policy-making, and environmental conservation in regions experiencing similar LULC dynamics.
- **Clarity**:
  The manuscript is **generally clear** and well-organized. The introduction, methods, and results are logically structured, and the use of figures and tables enhances understanding. However, there are instances where the text could be more concise, particularly in the results section where some information is repeated. Additionally, there are a few grammatical and typographical errors that need correction. The flow in some sections, especially the conclusion, could be improved for better readability.
- **ScientificRigor**:
  The **scientific rigor** is solid overall. The methods for LULC change detection (e.g., image classification and accuracy assessments) are appropriate and well-executed. The conclusions are largely supported by the data, although the discussion could delve deeper into the implications of the observed trends (e.g., deforestation, agricultural expansion, water scarcity). A more thorough analysis of the drivers behind these changes would enhance the manuscript’s scientific value.

**3. Providing Constructive Feedback**

- **Strengths**:
  - The use of multi-temporal satellite imagery and change matrices provides robust evidence of LULC changes over three decades.
  - The accuracy assessment is thorough, with overall accuracies consistently above 90%, indicating reliable classifications.
  - The study presents valuable insights into the ongoing environmental degradation in the SMW, which could inform policy and conservation efforts.
- **Areas for Improvement**:
  - **Accuracy Discussion**: Expand on why certain LULC classes (e.g., shrubland) had lower accuracy in earlier years, and discuss any classification challenges.
  - **Drivers of Change**: Provide more detailed analysis on the socio-economic or policy-driven factors contributing to the observed LULC changes. For example, how do population growth, agricultural practices, or government policies affect land-use patterns?
  - **Environmental Consequences**: Discuss the broader ecological implications of the observed trends (e.g., biodiversity loss, soil erosion, water scarcity) in more detail.
  - **Actionable Recommendations**: Make the recommendations in the conclusion more specific and actionable. For example, suggest specific reforestation techniques or alternative income sources for forest-dependent communities.
  - **Grammar and Formatting**: Proofread the manuscript to correct grammatical errors and ensure consistent formatting, particularly in the presentation of numbers and abbreviations.

**4. Checking for Ethical Issues**

- **Ethical Standards**:

There are no **apparent ethical concerns** with the research methodology or data collection. The study relies on satellite imagery for LULC classification, which does not involve human or animal subjects. The manuscript does not raise any issues related to research integrity or conflicts of interest.

- **Conflicts of Interest**:

The manuscript does not mention any conflicts of interest. If applicable, the authors should explicitly state whether there are any financial or personal conflicts that could influence the research. If no conflicts exist, this should be clearly mentioned in the manuscript.

**5. Structure of the Review**

- **Introduction**:
  The introduction provides a clear background on the importance of monitoring LULC changes, particularly in the context of forest degradation and agricultural expansion. However, it could benefit from a more explicit statement of the research question and objectives.
- **Methods**:
  The methodology is appropriate for the study’s objectives. The authors provide a detailed description of the satellite data used and the classification techniques. The accuracy assessment methods are robust, but the text could briefly mention any limitations of the classification approach.
- **Results**:
  The results are well-presented, with a clear explanation of the LULC changes over time. The use of tables and figures is effective, though some repetitive information could be condensed. The results would benefit from a more in-depth discussion of the underlying drivers of the observed changes.
- **Discussion**:
  The discussion ties the results to broader environmental issues, but it could be expanded to include a deeper analysis of the socio-economic drivers of LULC changes. The environmental consequences of deforestation, agricultural expansion, and water body reduction should be more thoroughly explored.
- **Conclusion**:
  The conclusion summarizes the key findings but lacks specificity in its recommendations. The authors should provide more actionable steps for mitigating the negative effects of LULC changes, such as specific reforestation techniques or policy interventions.

**Overall Recommendation**

This manuscript presents a valuable assessment of LULC changes in the Sangu-Matamuhuri Watershed over three decades. It provides important insights into forest degradation and agricultural expansion, which have significant environmental and socio-economic implications for the region. The methodology is sound, and the results are well-supported by the data. However, the manuscript would benefit from:

- A more detailed discussion of the drivers behind LULC changes.
- A stronger emphasis on the environmental and socio-economic consequences of these changes.
- More specific and actionable recommendations for addressing the observed trends.
- Minor revisions for grammar, clarity, and flow.

**Recommendation**:

- **Minor revisions required** before acceptance for publication. The manuscript is scientifically rigorous but requires some improvements in the discussion, conclusion, and presentation of the findings.
