# Supplementary material for: Spatiotemporal pattern of land use and land cover changes of upper Sangu-Matamuhuri Watershed in the South-Eastern Bangladesh
Source: PLoS One. 2025 Jul 2;20(7):e0327284. doi: 10.1371/journal.pone.0327284 (PMC12221057; doi:10.1371/journal.pone.0327284)
Supplement: Attachment — Submitted filename: Response to Reviewers.docx [file pone.0327284.s009.docx]

**Reference: *Spatiotemporal pattern of land use and land cover changes of upper Sangu-Matamuhuri watershed in the South-Eastern Bangladesh***

Responses of the authors to the comments of the Editors and Reviewers

**Response to editor’s comments:**

1. **Please ensure that your manuscript meets PLOS ONE's style requirements, including those for file naming. The PLOS ONE style templates can be found at**

**https://journals.plos.org/plosone/s/file?id=wjVg/PLOSOne_formatting_sample_main_body.pdf and**

**https://journals.plos.org/plosone/s/file?id=ba62/PLOSOne_formatting_sample_title_authors_affiliations.pdf**

**Authors: PLOS One Manuscript Body Formatting Guidelines and Title, Authors, Affiliations Formatting Guidelines have been followed accordingly during manuscript preparation.**

2. **We note that Figures 1 and 4 in your submission contain [map/satellite] images which may be copyrighted. All PLOS content is published under the Creative Commons Attribution License (CC BY 4.0), which means that the manuscript, images, and Supporting Information files will be freely available online, and any third party is permitted to access, download, copy, distribute, and use these materials in any way, even commercially, with proper attribution. For these reasons, we cannot publish previously copyrighted maps or satellite images created using proprietary data, such as Google software (Google Maps, Street View, and Earth). For more information, see our copyright guidelines: http://journals.plos.org/plosone/s/licenses-and-copyright.**

**We require you to either (1) present written permission from the copyright holder to publish these figures specifically under the CC BY 4.0 license, or (2) remove the figures from your submission:**

**a. You may seek permission from the original copyright holder of Figures 1 and 4 to publish the content specifically under the CC BY 4.0 license.**

**We recommend that you contact the original copyright holder with the Content Permission Form (http://journals.plos.org/plosone/s/file?id=7c09/content-permission-form.pdf) and the following text:**

**“I request permission for the open-access journal PLOS ONE to publish XXX under the Creative Commons Attribution License (CCAL) CC BY 4.0 (http://creativecommons.org/licenses/by/4.0/). Please be aware that this license allows unrestricted use and distribution, even commercially, by third parties. Please reply and provide explicit written permission to publish XXX under a CC BY license and complete the attached form.”**

**Please upload the completed Content Permission Form or other proof of granted permissions as an ""Other"" file with your submission.**

**In the figure caption of the copyrighted figure, please include the following text: “Reprinted from [ref] under a CC BY license, with permission from [name of publisher], original copyright [original copyright year].”**

**b. If you are unable to obtain permission from the original copyright holder to publish these figures under the CC BY 4.0 license or if the copyright holder’s requirements are incompatible with the CC BY 4.0 license, please either i) remove the figure or ii) supply a replacement figure that complies with the CC BY 4.0 license. Please check copyright information on all replacement figures and update the figure caption with source information. If applicable, please specify in the figure caption text when a figure is similar but not identical to the original image and is therefore for illustrative purposes only.**

**The following resources for replacing copyrighted map figures may be helpful:**

**USGS National Map Viewer (public domain): http://viewer.nationalmap.gov/viewer/**

**The Gateway to Astronaut Photography of Earth (public domain): http://eol.jsc.nasa.gov/sseop/clickmap/**

**Maps at the CIA (public domain): https://www.cia.gov/library/publications/the-world-factbook/index.html and https://www.cia.gov/library/publications/cia-maps-publications/index.html**

**NASA Earth Observatory (public domain): http://earthobservatory.nasa.gov/**

**Landsat: http://landsat.visibleearth.nasa.gov/**

**USGS EROS (Earth Resources Observatory and Science (EROS) Center) (public domain): http://eros.usgs.gov/#**

**Natural Earth (public domain): http://www.naturalearthdata.com/**

**Authors: Figure 1 in our submission is basically prepared by authors in earlier publication (Title: ‘Streamflow characteristics of Sangu-Matamuhuri watershed in the Southeastern part of Bangladesh’, Published in Heliyon journal, Volume 9, Issue 3, March 2023, e14559, Link:** [**https://www.sciencedirect.com**](https://www.sciencedirect.com)**/science/article/pii/S2405844023017668.** [**https://www.cell.com/heliyon/pdf/S2405-8440(23)01766-8.pdf)**](https://www.cell.com/heliyon/pdf/S2405-8440(23)01766-8.pdf))**.**

**Figure 4 in our submission is also prepared by authors. But when submitted earlier for publication in other Journal**, **it was unauthorizely published as a preprint by Research Gate, Research Square.**

3. **We note you have included a table to which you do not refer in the text of your manuscript. Please ensure that you refer to Table 2 in your text; if accepted, production will need this reference to link the reader to the Table.**

**Authors:** **Table 2. LULC classification scheme used for the study was modified from ADB, 2001. It was already mentioned in the Text (Page 11, Line 224, 230) and reference (Page 32, Line 682) in accordance with guideline. Please see page 11, line 216, 221.**

**Additional Editor Comments:**

Your manuscript entitled "Spatiotemporal changing pattern of land use and land cover of Sangu-Matamuhuri watershed in the South-Eastern part of Bangladesh", which you submitted to Cogent Food & Agriculture, has now been reviewed.

**Authors: The Author didn’t submit manuscript titled ‘"Spatiotemporal changing pattern of land use and land cover of Sangu-Matamuhuri watershed in the South-Eastern part of Bangladesh" to ‘Cogent Food & Agriculture’.**

**Responses to Reviewers comments:**

**Reviewer #1:** The manuscript needs some conceptual and factual corrections and clarifications. The details attached in Microsoft Word document using track changes functionality. Therefore, the authors should incorporate the comments and suggestions attached.

**Authors: All conceptual and factual corrections and clarifications as mentioned in Microsoft Word document of the manuscript using track changes functionality are accomplished and the comments and suggestions attached therein are also incorporated in the manuscript.**

**A marked-up copy of our manuscript that highlights changes made to the original version is uploaded as a separate file labeled 'Revised Manuscript with Track Changes'.**

**Major Comments:**

**Summary of Key Findings:**

**While the conclusion summarizes the key findings effectively, I recommend expanding the discussion to link these findings to broader implications, such as climate change and biodiversity loss. This will emphasize the urgency of the observed changes.**

**Authors: The key findings of the study are elaborately described in conclusion part with actionable steps or recommendations for broader implications.**

**Ecosystem Services:**

**The manuscript mentions threats to ecosystem services but could elaborate on how LULC changes impact services like water regulation and soil conservation. This connection would provide a more comprehensive understanding of the ecological consequences.**

**Authors: According to editor’s advice, impact of LULC changes on ecosystem services including water regulation and soil conservation are elaborated for better understanding of the ecological consequences in the discussion chapter.**

**Recommendations for Future Actions:**

**I encourage you to provide specific, actionable recommendations for reforestation and sustainable land management practices. Including examples such as agroforestry techniques or alternative livelihoods for forest-dependent communities would enhance the practicality of your suggestions.**

**Authors: Specific and actionable recommendations for reforestation and sustainable land management practices including examples such as agroforestry techniques or alternative livelihoods for forest-dependent communities are provided in the conclusion chapter.**

**Water Resources and Community Impacts:**

**Expand the discussion on the socio-economic impacts of water scarcity, particularly how it affects agricultural productivity and local communities. Addressing potential strategies for water conservation would also be beneficial.**

**Authors: Socio-economic impacts of water scarcity with particular effect upon agricultural productivity and local communities are elaborately discussed in the discussion chapter. Potential strategies for water conservation are also addressed.**

**Urgency and Long-Term Outlook:**

**Strengthening the sense of urgency regarding LULC changes will underscore the importance of immediate intervention. Discussing long-term risks associated with continued degradation can help frame the necessity for comprehensive land-use planning.**

**Authors: Short and long-term strategies in order to combat risks associated with continued degradation due to LULC changes are addressed for comprehensive land-use planning.**

**Minor Comments:**

**Clarity and Flow: Improve transitions between points in the conclusion to create a cohesive narrative.**

**Authors: Transitions between points in the conclusion chapter are improved**.

**Consistency in Data Presentation: Reduce redundancy by summarizing key findings without repeating exact figures from previous sections.**

**Authors: The key findings of the study are elaborately described for broader implications. Repetitions of exact figures from previous sections are avoided.**

**Technical Terminology: Avoid abbreviations without explanation to ensure accessibility for all readers.**

Author: Abbreviations used throughout this study are explained accordingly.

**Typographical and Grammatical Issues: Proofread the manuscript for grammatical errors and awkward phrasing.**

**Authors: Proof reading has been performed by native speaker and conducted necessary corrections.**

**Ethical Considerations:**

**I found no ethical concerns regarding research methodology or data collection. The manuscript does not raise issues of dual publication or conflict of interest; if applicable, please explicitly state this.**

**Author: This article titled “Spatiotemporal pattern of land use and land cover of upper Sangu-Matamuhuri watershed in the South-Eastern Bangladesh” is an original research performed by authors. All ethical practices have been followed in the writing of this manuscript.**

--------------------------0-------------------------------
