# Supplementary material for: Spatiotemporal pattern of land use and land cover changes of upper Sangu-Matamuhuri Watershed in the South-Eastern Bangladesh
Source: PLoS One. 2025 Jul 2;20(7):e0327284. doi: 10.1371/journal.pone.0327284 (PMC12221057; doi:10.1371/journal.pone.0327284)
Supplement: Attachment — Submitted filename: Response to Reviewers_R2.docx [file pone.0327284.s010.docx]

**Reference: *Spatiotemporal pattern of land use and land cover changes of upper Sangu-Matamuhuri watershed in the South-Eastern Bangladesh***

Responses of the authors to the comments of the Academic Editor and Reviewers

**Response to editor’s comments:**

**Journal Requirements:**

1. Please review your reference list to ensure that it is complete and correct. If you have cited papers that have been retracted, please include the rationale for doing so in the manuscript text, or remove these references and replace them with relevant current references. Any changes to the reference list should be mentioned in the rebuttal letter that accompanies your revised manuscript. If you need to cite a retracted article, indicate the article’s retracted status in the References list and also include a citation and full reference for the retraction notice.

**Authors:** According to PLOS One submission guidelines, reference list is reviewed and changes made to the reference list according to citation in the text are mentioned as follows:

Reference numbers are corrected according to citation in the text:

- Reference number 21-27 were wrongly numbered in the text and so corrected as 6-12	according to text.	- Reference number 13 is newly included.	- Reference number 28, 29 are replaced as 14 and 15 according to text.	- Reference number 7 is replaced as 16 according to text.	- Reference number 31-37 are replaced as 17-23 respectively according to text.	- Reference number 6 is replaced as 24 according to text.	- Reference number 8-20 are replaced as 25-37 according to text.	- Reference number 30, 40, 43 are subtracted due to their duplication.	- Reference number 41-42 are replaced as 40-41 according to text.	- Reference number 42-43 are newly included according to text.	- Reference number 66 is subtracted due to its non-relevancy in the text.	- Reference number 67 is replaced as 66 according to text.	- Reference number 68, 69 are replaced as 43, 67 respectively according to text.	- Reference number 68, 69 and 70 are newly included.	-

**Additional Editor Comments:**

2. While the conclusion summarizes the key findings effectively, expanding the discussion to link these findings to broader implications, such as climate change and biodiversity loss are required from the authors.

**Authors**: In the discussion section (Line 419-425, 441-449, 479-487), all key findings are broadly discussed with their implications including climate change and biodiversity loss.

3. The manuscript mentions threats to ecosystem services but could elaborate on how LULC changes impact services like water regulation and soil conservation is remains unclear.

Authors: Impact of LULC changes upon ecosystem services including water regulation and soil conservation are elaborately discussed in the discussion section (Line 419 – 425) as mentioned below:

‘However, this LULC change has a large negative impact upon watershed ecosystem services such as water regulation, soil conservation, and carbon sequestration. Deforestation disrupts the natural water cycle, leading to increased surface runoff, soil erosion, and reduced water availability; exposes the soil, making it vulnerable to erosion and degradation; releases the stored carbon back into the atmosphere, contributing to climate change; and also reduces the capacity of forests to absorb carbon dioxide in the future [34, 58, 69, 70].’

4. Expand the discussion on the socio-economic impacts of water scarcity, particularly how it affects agricultural productivity and local communities. Addressing potential strategies for water conservation would also be beneficial.

Authors: Socio-economic impacts of water scarcity relating to agricultural productivity and local communities are broadly discussed in the discussion section (Line 441-445) as mentioned below:

‘This situation makes the local communities vulnerable to natural disasters, such as flash floods, landslides in one side and droughts on the other. It was reported that people have to travel long distances to fetch drinking water during dry season [66]. Their livelihoods including agriculture, forestry, fishing are severely affected which leads them towards economic hardship and social instability [34, 38, 66].’

Potential strategies for water conservation are also addressed (in line 445-449) as follows:

‘Proper upland water management including community led site specific soil and water conservation works such as reforestation measures with native tree species of high water retention capacity, composting, mulching, intercropping, making percolation pond, constructing mini-check dam, etc. should be ensured to overcome this adverse situation in the study area.’

5. Discussing long-term risks associated with continued degradation can help frame the necessity for comprehensive land-use planning.

Authors: In order to frame the necessity for comprehensive land-use planning, long-term risks associated with continued degradation are discussed in the discussion section (Line 479 - 482) as follows:

‘Overpopulation and expansion of unplanned agriculture in the study area causes gradual deforestation which ultimately resulting biodiversity loss, soil degradation and significant contribution to global climate change, etc. [42, 43]. Continuation of this scenario will result in more degraded land in the near future.’

6. Clarity and Flow: Improve transitions between points in the conclusion to create a cohesive narrative.

Authors: In order to create a cohesive narrative, transitions between points in the conclusion are improved.

7. Consistency in Data Presentation: Reduce redundancy by summarizing key findings without repeating exact figures from previous sections.

Authors: Key findings are summarized without repeating exact figures from previous sections.

8. Technical Terminology: Avoid abbreviations without explanation to ensure accessibility for all readers.

Authors: Abbreviations are explained wherever needed.

9. Typographical and Grammatical Issues: Proofread the manuscript for grammatical errors and awkward phrasing.

Authors: Manuscript are proof red for grammatical errors and awkward phrasing.

10. The present study is at a watershed level. Hence, your conclusion should reflect the implications of the land use change on watershed management. So, you should add a paragraph or two in this regard.

Authors: A paragraph regarding implications of the land use change on watershed management is added in the conclusion section (520 – 527) as follows:

‘Hence, in order to mitigate the negative effects of LULC changes, proper management of watershed resources is required. An integrated watershed management program should be undertaken considering all biophysical, socio-economic, environmental and institutional issues beyond as well within the area. Identifying degraded micro-watershed, adequate forest restoration measures including enrichment planting, assisted natural regeneration, site specific soil and water conservation works, organizing stakeholder consultation meeting with all institutions and leaders and training of community people on resilient livelihood skill development should be carried out immediately in the watershed area.’

11. Forest degradation refers to the decline in forest quality, health, and functionality due to various factors. In your study you focused on land use change. So, change this to ‘loss of forest land’.

Authors: The term ‘Forest degradation’ is changed to ‘loss of forest land’.

12. While revising your submission, please upload your figure files to the Preflight Analysis and Conversion Engine (PACE) digital diagnostic tool, <https://pacev2.apexcovantage.com/>.

Authors: All figure files are uploaded to the Preflight Analysis and Conversion Engine (PACE) digital diagnostic tool, https://pacev2.apexcovantage.com/.

**Responses to Reviewers comments:**

**Reviewer #1:** The authors of this article have included all the comments and suggestions, which I raised in the early phase of reviewing the manuscript. The only issue I have is that they declared that they did not get fund for conducting the project, but they eventually acknowledged Bangladesh Forest Department. What kind of support did they get from the institution?

Authors: Yes. Authors did not get any fund from Bangladesh Forest Department. But authors got technical support from Bangladesh Forest Department in terms of performing field studies in the Sangu-Matamuhuri Reserve Forests area which belongs to Bangladesh Forest Department, data collection, etc.

-------------------------
